# Editorial: Pain in the older adult patient

**DOI:** 10.3389/fmed.2025.1642862

**Published:** 2025-06-24

**Authors:** Luca Tagliafico, Rose Penfold, Sofia Duque, Stefano Cacciatore

**Affiliations:** ^1^Department of Internal Medicine and Medical Specialties (DIMI), University of Genoa, Genoa, Italy; ^2^Edinburgh Delirium Research Group, Ageing and Health, Centre for Population Health Sciences, Usher Institute, University of Edinburgh, Edinburgh, United Kingdom; ^3^Advanced Care Research Centre, Usher Institute, University of Edinburgh, Edinburgh, United Kingdom; ^4^Faculty of Medicine, Preventive Medicine and Public Health Institute, University of Lisbon, Hospital Cuf Descobertas, Lisbon, Portugal; ^5^Department of Geriatrics, Orthopedics and Rheumatology, Università Cattolica del Sacro Cuore, Rome, Italy

**Keywords:** chronic pain, frailty, aged, physical performance, pain assesment, pain management (MeSH), multimorbidity

**Figure 1:**
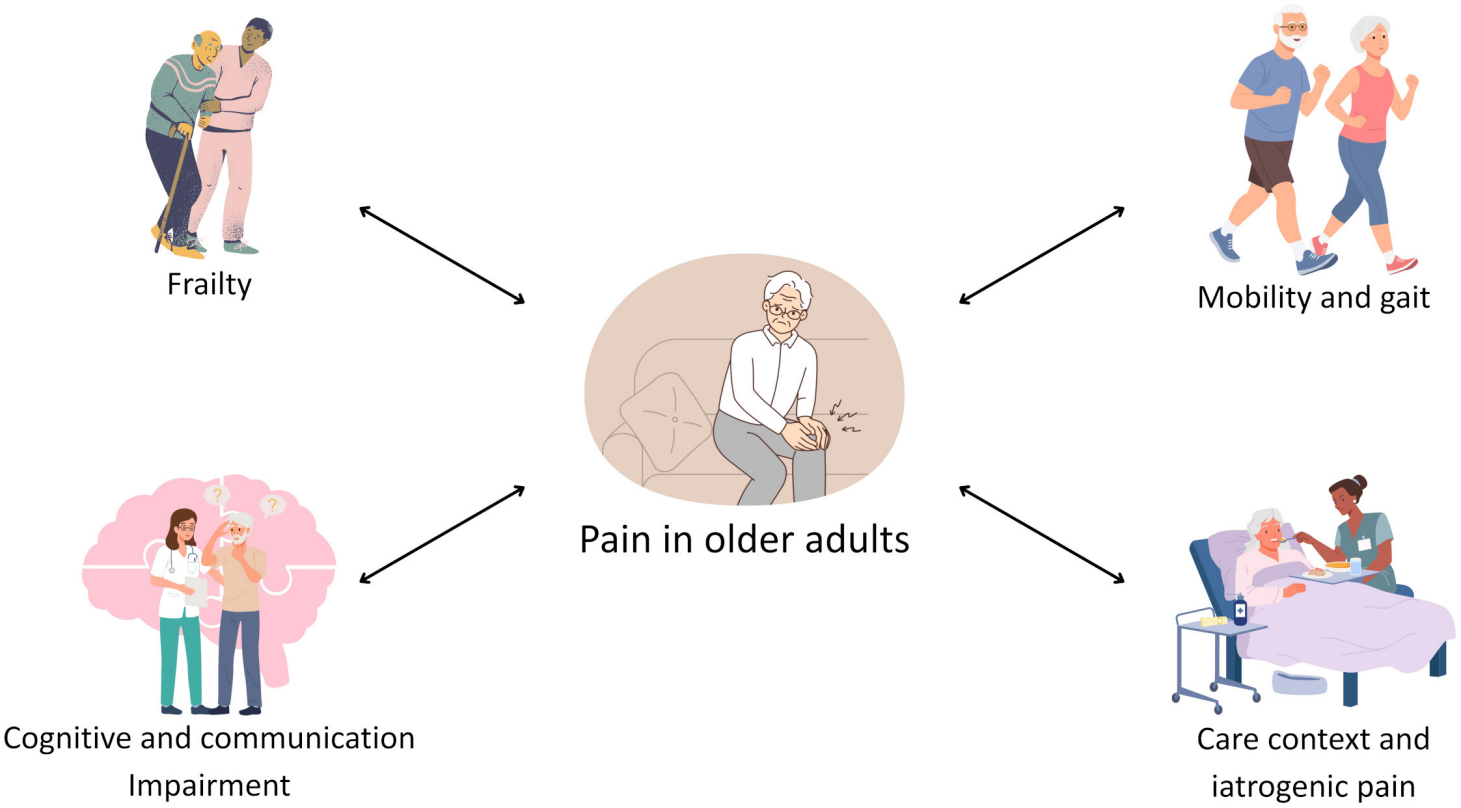
The interplay between pain, frailty, and functional outcomes in older adults.

Pain is defined as “an unpleasant sensory and emotional experience associated with, or resembling that associated with, actual or potential tissue damage” (1). The prevalence of chronic pain in older adults is a significant public health concern. Community-dwelling individuals are affected to a considerable extent, with prevalence rates reaching up to 50%. This figure increases substantially for long-term care facility residents, with up to 80% of this demographic experiencing chronic pain. The occurrence of chronic pain is closely related to age-related conditions, including neurodegenerative, musculoskeletal, and vascular diseases, as well as arthritis and osteoarthritis (2). Given its profound impact on outcomes and interplay with geriatric syndromes including frailty, poor mobility, and cognitive decline (Figure 1), pain represents a critical marker of health status in this population (3).

Despite its importance, pain is still under recognized and undertreated in older adults, especially in frail individuals or in those with cognitive impairment, in whom it may present atypically (4). Additional factors, such as sensory decline, language barriers, or socioeconomic vulnerability, may further obscure clinical detection (4). This underrecognition may result in the progression of acute or chronic pain episodes into complications including delirium, hospitalization, and ongoing functional decline (5).

The aim of this Research Topic was to invite articles which explore the epidemiology and management of pain in older adults, focusing in particular on the interplay with frailty and multimorbidity. Together, the articles included in this Research Topic provide complementary perspectives on the clinical and functional implications of pain in later life.

Starting with the article by Zhong et al., a two-sample Mendelian randomization approach was used to assess the bidirectional association between pain and frailty. Their findings suggest that genetic predisposition to frailty increases the risk of various types of pain, particularly joint, limb, and low back pain, while genetic vulnerability to pain, in turn, raises the likelihood of developing frailty. This underscores the necessity of a dual approach to prevention and management, recognizing that pain as both a consequence and a potential driver of frailty.

In a complementary perspective, Madsalae et al., evaluated gait changes in older adults with chronic neck pain, focusing on speed and symmetry during walking with head movements. They observed that chronic neck pain significantly impairs dynamic balance, suggesting that even localized pain syndromes can have broad repercussions on mobility and fall risk. This highlights the importance of early identification and management of specific pain subtypes to preserve functional status.

Tagliafico et al. shifted the focus to non-communicative older adults, examining pain expression beyond the context of dementia. Their review highlights the limitations of current observational pain assessment tools: most of them are validated in healthy older adults, some in patients with neurocognitive disorders such as dementia, and very few in conditions conferring sensory impairments such as Parkinson's disease or post-stroke aphasia. The authors advocate for the development of more nuanced instruments tailored to a wider range of sensory and communicative impairments.

The contribution by Magi et al. addresses the overlooked issue of procedural pain during routine nursing care. Despite their routine nature, procedures like venipuncture or catheterization remain insufficiently addressed in older inpatients. The authors emphasize the need for multidisciplinary, patient-centered strategies that integrate pharmacologic and non-pharmacologic approaches, placing nurses at the forefront of pain management in this context.

Finally, Li et al. present the protocol of a randomized controlled trial assessing the combined effect of Chinese herbal therapy and pulsed electromagnetic fields in older adults with knee osteoarthritis. The trial will explore both symptom relief and functional improvement, offering a valuable contribution to the integration of complementary therapies into mainstream geriatric pain management.

In conclusion, managing pain in older adults remains a major clinical and public health challenge, made more complex by cognitive, functional, and communicative barriers. The studies featured in this Research Topic collectively underscore the importance of nuanced assessment strategies, interdisciplinary interventions, and a deeper understanding of the bidirectional relationship between pain and other geriatric syndromes. Future research should aim not only to refine detection and treatment, but also to integrate pain management into broader frameworks of healthy aging and personalized care.
